# Niemann-Pick Disease: An Approach for Diagnosis in Adulthood

**DOI:** 10.7759/cureus.4767

**Published:** 2019-05-28

**Authors:** Bonell Patiño-Escobar, Maria H Solano, Laura Zarabanda, Claudia P Casas, Carlos Castro

**Affiliations:** 1 Hematology, San Jose Hospital – University Foundation of Health Sciences, Bogotá, COL; 2 Seedbed of Research Program, San Jose Hospital – University Foundation of Health Sciences, Bogotá, COL; 3 Epidemiology and Public Health, San Jose Hospital – University Foundation of Health Sciences, Bogotá, COL

**Keywords:** niemann-pick disease, type b, lysosomal acid lipase deficiency, rare disease, splenomegaly

## Abstract

Niemann-Pick (NP) disease is a rare, autosomal recessive disorder characterized by visceromegaly and neurological alterations due to the excessive storage of lipids, sphingomyelin, and cholesterol. It commonly affects the child population, and only 6% of it occurs in the adult population. Type A is classified as the acute form, type B is the latest and with the best prognosis, and type C is characterized by neurological alteration. The diagnosis is based on enzymatic tests and genetic sequencing, with the latter being the diagnostic confirmation test. No specific treatment exists for this entity, although some patients with NPC type C may benefit from pharmacological treatment with miglustat.

The objective of this paper is to describe the clinical characteristics of a grown patient with Niemann-Pick diagnosis type B.

This article reports the case of a 55-year-old adult patient with a three-year clinical history consisting of splenomegaly and hematological disorders, without neurological symptoms ruling out frequent pathologies. Type B NP disease is diagnosed by a mutation in the sphingomyelin phosphodiesterase 1 (SMPD1) gene. The patient was receiving multidisciplinary support treatment.

Although NP disease is a rare disease according to the literature, it is important to consider this group of disorders as a differential diagnosis, when other more common pathologies have been ruled out in patients with isolated splenomegaly and thrombocytopenia

## Introduction

Lysosomal storage diseases are characterized by an enzymatic deficiency that involves the main function of lysosomes. Currently, there are 50 different kinds of heritage metabolic diseases [1]. Niemann-Pick (NP) disease, within this group, is an autosomal recessive disease. It is defined as the acid sphingomyelinase enzyme deficiency (SED), producing an alteration in the sphingomyelin and lipids deposits, thus causing a structural and functional change in the cell and viscera tissue [2]. The global prevalence is estimated between four and six cases per 1,000,000 of inhabitants [3] and is more frequent in the ascendant Jewish population. NP is diagnosed in 80% of cases under the age of 16 years, in 14% after 20 years of age, and extremely rare in older age, depending on the subtype [4-5].

Albert Niemann reported the first case in 1914, but in 1927, Ludwig Pick presented it as a different disease, marking its histological difference from the Gaucher disease [6]. The NP disease is classified based on organ involvement and enzymatic alteration. There are four sub-types (Types A, B, C, D), depending on the organ involved, age, and symptoms, with subtype B being the most frequent. With regard to enzymatic deficiency, in 1966, it was demonstrated that the acid sphingomyelinase enzyme deficiency (SED) in NP types A and B, but not in NP types C and D. Due to the above-mentioned, the four types of NP disease may be classified into two categories regarding the enzymatic deficiency: Type I is characterized by low acid sphingomyelinase levels seen in NP types A and B. In type II, a defect in low-density lipoprotein (LDL) transportation is seen in NP types C and D. The clinical presentation is related to visceromegaly and central nervous system involvement [2]. Diagnosis is based on clinical assessment and diagnostic tests, such as enzymatic quantification, fibroblast culture (Type C), and molecular biology techniques for identification (NPC1, NPC2, and SMPD-1 gene mutation identification). Later, a more specific genetic test should be made in order to obtain a specific diagnosis [7]. Treatment typically targets general symptom management: nevertheless, when there are neuropsychological disturbances, miglustat indication has shown a tendency toward delaying the neuronal degeneration in NP disease type C [2,8-9].

In Colombia, an orphan disease is defined as “A disease chronically debilitating, grave, life-threatening, and include rare diseases such as ultra-orphan diseases and forgotten diseases.” These diseases have more than 1900 pathologies [10-11], which impact the health system considerably, especially those considered high-cost diseases. The identification of such low-frequency diseases becomes a challenge, and hence the importance of recognizing the unique clinical presentation that allows the most appropriate identification and treatment. The main objective of this report is to describe the clinical features of a patient with NP disease type B diagnosed in old age.

## Case presentation

We present the case of a 55-year-old male patient, who was administered because of a five-year history of abdominal pain localized in the upper left quadrant and hypogastrium, with associated symptoms of weight loss and night sweat that emerged six months prior to his consultation. Looking back into his medical history, the patient had first-grade relatives who suffered pancreatic cancer. He had undergone splenectomy three years before, due to splenomegaly of undetermined cause, with a relief of the symptoms as mentioned above. The patient had no abnormalities on hematology, no visceromegaly, no neurologic disturbances, and no enzymatic alteration on laboratory tests that could lead to a diagnosis of lysosomal storage disease. Further laboratory tests were ordered in order to rule out Gaucher disease vs. Niemann Pick, taking into account the discordance between the enzymatic analysis and the clinical presentation. After a more detailed anamnesis, thrombocytopenia was documented at another institution in the past, which was fluctuant and variable (less than 100.000 cell/mL) and a pathology report from the spleen (Table 1).

**Table 1:** Laboratory test performed for the patient HIV: human immunodeficiency virus; HCV: hepatitis C virus; FTA-ABS: fluorescent treponemal antibody absorption; EBV: Epstein–Barr virus; TGO: glutamic oxalacetic transaminase; TGP: glutamic pyruvic transaminase; PT: prothrombin time; INR: international normalized ratio; PTT: partial thromboplastin time; LDL: low-density lipoprotein; HDL: high-density lipoprotein

Laboratory test	Description / Values
Spleen and lymph node histopathology description	Macroscopic: Spleen of 410 g of weight. Microscopic: Wide expansion of red pulp by several macrophages, with wide and foamy cytoplasm, negative for PAS stain. Few iron deposits. Secondary splenomegaly due to red pulp occupation by ceroid macrophages and lymph node with paracortical lymphoid hyperplasia Immunohistochemistry: Negative for neoplasm.
Bone marrow biopsy	Cellularity: 50%. Myeloid-erythroid relation: 1:1. Platelets cells: Present. Little platelet cumulus. Erythroid cells: Normal. Myeloid cells: Mature predominance. Lymphoid cells: 11%, monocytes: 2%, plasmocytes: 1%, Blast cells: 1%. Occasional histiocytes. No neoplastic involvement. There are 10% of histiocytes with features of foamy cells, in an interstitial distribution, which they are negative for PAS staining. Findings related to NP disease.
Beta-glycosidase	16.6 mmol/mh (6–9 mmol/mh)
Chitotriosidase	47.8 nmol/hr/ml (< 96.7 nmol/hr/ml )
Infectious: HIV/HCV-as/FTA-ABS/EBV	Negative
Liver function test: TGO/TGP/Direct Bilirubin/ indirect Bilirubin/Albumin	24 U/L ( 15–37 U/L ) / 35 U/L ( 30–65 U/L)/ 0.25 mg/dl (0.00–0.30 mg/dl) / 0.6 mg/dl ( 0.00–0.70 mg/dl) / 4.48 g/dl ( 3.4–4.0 g/dl)
Coagulation: PT/INR/PTT	11.6 seg (11.3 seg) / 1.03 seg / 26.3 seg. (25.9 seg.)
Karyotype	46XY (12), No chromosome alterations
Lipid profile: HDL/LDL/Total Cholesterol/Triglycerides	30 mg/dl ( > 40 mg/dl) / 112.3 mg/dl ( < 130 mg/dl) / 191 mg/dl (< 190 mg/Dl) / 233 mg/dl (30-200 mg/dl)
Gene mutation sequencing (centogen)	Compatible with NP disease type A/B, caused by mutation detected through SMPD1 gene sequencing

**Figure 1:**
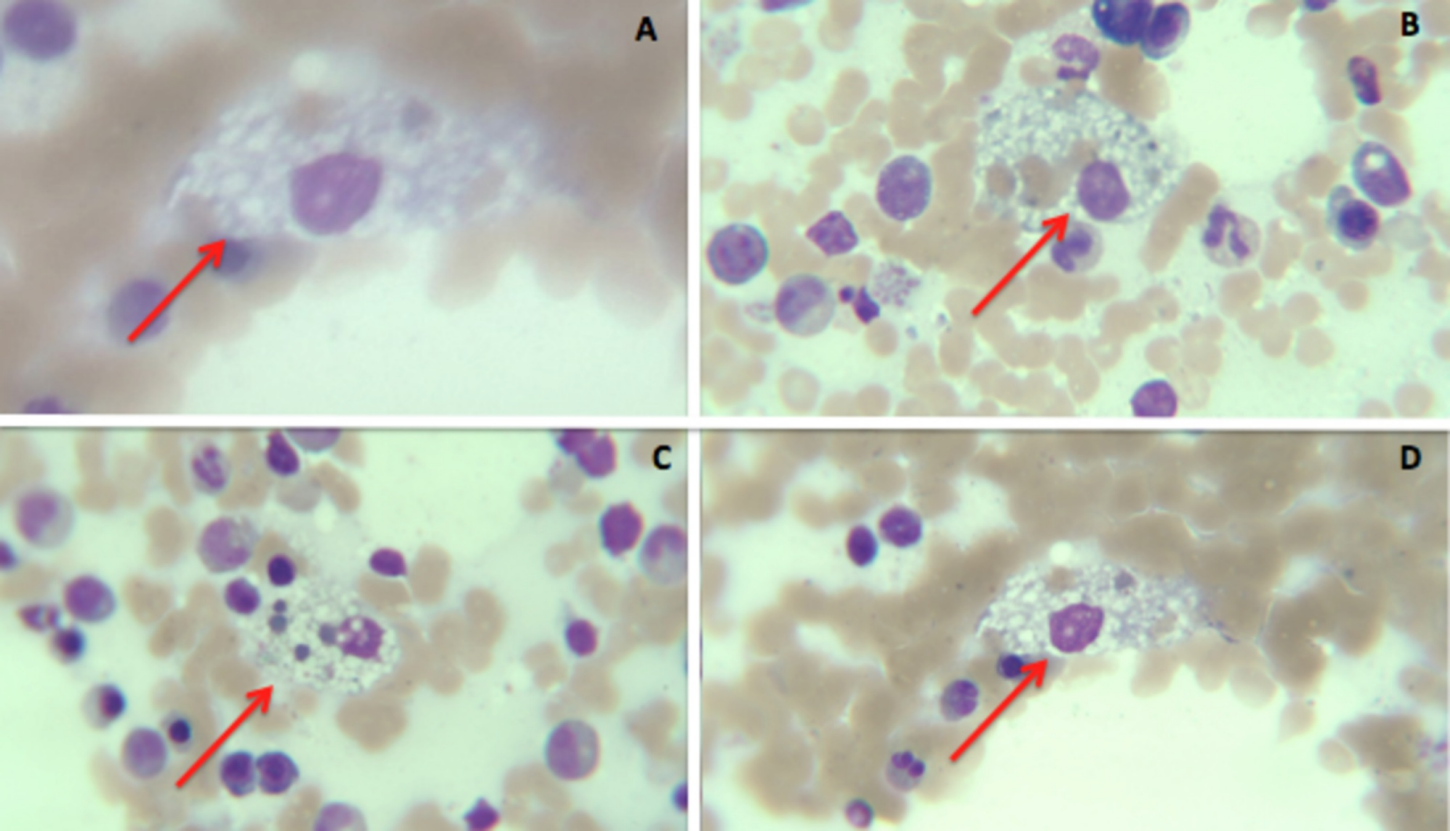
Myelogram where the foam cells (Niemann-Pick cells: soap-suds appearance) were found

After the first evaluation of the hematology results, genetic sequencing was performed, an NP disease was diagnosed (Table 1), a myelogram was done (Figure 1), and flow cytometry was performed. Due to those reports and the clinical features, the NP disease was classified as type B. The patient was assessed by neurology, documenting a headache with features such as migraine and normal angiography. Propanolol 40 mg in a day was ordered for prophylactic management. Furthermore, the patient underwent a neuropsychological assessment to establish a cognitive profile, to identify any failure in self-monitoring, planning, organization, and impulsivity. In accordance with the above, and based on the disease classification, there was no indication of miglustat in this case. Therefore, follow-up and symptom monitoring every six months were decided, including evaluating platelets count and liver and pulmonary test function.

## Discussion

Orphan diseases are very rare, affecting a small group of people who require highly complex diagnoses. They are life-threatening and cause weakness in the long term [12]. In Colombia, according to SISPRO (Integral Information System of Social Protection) reports, the rate of orphan disease is estimated to be 10 to 28 cases per 100.000 inhabitants [13], making them rare diseases that are less frequently diagnosed in adulthood [1,14]. Among those diseases are the lysosomal storage diseases, which originate from a genetic alteration in the enzymes and proteins in charge of macromolecule hydrolysis derived from cellular catabolism.

Ninety-six percent of those diseases have a recessive autosomal heritage origin, such as the NP disease case [1,12]. NP types A and B have an incidence of 1 in 250.000 people, whereas the incidence of type C is 1 in every 120,000 living births [15-16]. It is sub-divided depending on the enzymatic alteration type, hence, when acid sphingomyelinase activity (ASM) is null, the enzyme coded by the gene localized on chromosome 11 (p15.1 to p15.4) [17], sphingomyelin phosphodiesterase 1 (SMPD-1), causes a buildup of sphingomyelin, inducing cell death and loss of organ functioning, producing NP disease types A and B [16,18]. In this case, it was possible to determine a relationship between SMPD-1 gene mutation and functional and structural alterations of the spleen, which was clinically evident as splenomegaly in adulthood, a situation that is less frequent due to its presentation being almost always in childhood [7,16]. The clinical presentation may be unspecific and affects several systems, producing high mortality rates in the pediatric population [1,19]. In this case report, we found a late beginning of the disease with signs and symptoms similar to splenomegaly, variable thrombocytopenia from hypersplenism [20] and no pulmonary symptoms, which usually are described in these kinds of patients. Symptoms were nonspecific, though they could be frequent in other kinds of diseases and depend on the penetrance of the disease, as seen from the results of the enzymatic test whose results may have low specificity. In addition, there was a lack of family history; all these are useful for diagnosis. Table 2 shows the signs and symptoms of every NP disease type [3,15].

**Table 2:** Signs, symptoms and other features of every Niemann-Pick disease type ASM: acid sphingomyelinase; LDL: low-density lipoprotein

System / Organ	Type A	Type B	Type C
Age of onset, genotype	Child population (Preschool). Mean of time to death: 27 months. Related to SMPD-1 gene with p.R498L, p.L304P y p.P333Sfs, 52 variants ASM deficiency	Child population/adulthood survival to adult age, death due to a progressive liver or pulmonary disease. Related to p.ΔR610, p.P323A, p.P330R y p.W393G variants. ASM deficiency	Child population/adulthood. Related to NPC1 y NPC2 genes, more than 352 mutations have been reported. Alteration of LDL transportation
Central nervous system	Mild hypotony, loss of deep tendon reflexes: dysphagia. Progressive neurodegeneration.	No neurodegeneration	Slow neuronal degeneration (ataxia, dystonia, and cognitive impairment). Psychomotor delay, central hypotony, hypoacusia. Progressive dysarthria. Seizures crisis
Ophthalmologic	30% of patients have a cherry red spot in the retina from 6 months of age	Ocular anomalies due to neuronal storage in some patients in the absence of neurologic disease	Supranuclear paralysis of vertical gaze
Gastrointestinal	Hepatosplenomegaly from 3 months of age. Jaundice emesis	Hepatosplenomegaly. Usually, splenomegaly onset is before hepatomegaly Portal hypertension, cirrhosis, ascites. Abnormal liver function test. Diarrhea	Hydrops hepatosplenomegaly. Persistent fetal ascites. Prolonged cholestasis. Youth and adult with only splenomegaly
Pulmonary	Frequent and recurrent respiratory infections: Aspiration pneumonia Interstitial lung disease	Interstitial lung disease. Restrictive features	Respiratory insufficiency
Cardiac	No abnormalities	Valvular heart disease. Mixed dyslipidemia. Coronary disease in early onset.	No heart involvement
Musculoskeletal	Delayed growth with low weight and height, producing slow skeletal maturation	Polyarthralgias. Bone density reduction with a high risk of pathologic fractures. Delayed bone maturation. Osteoporosis	No musculoskeletal involvement
Hematological	No hematological involvement or mild thrombocytopenia	Thrombocytopenia with a tendency of bleeding	No hematological involvement
Treatment	Liver transplantation (controversial) support	Bone marrow transplantation (controversial) support	Miglustat (controversial)

Genetic sequencing is the gold standard diagnostic test for the confirmation of NP disease. In this case, there was SMPD1 gene mutation information, which later confirmed the presence of NP cells in the bone marrow aspiration sample [17]. It is necessary to determine the enzymatic activity or proteins of acid sphingomyelinase (ASM) in leukocytes from peripheral blood, found in low levels (1%-0%) in patients with NP disease types A or B or by a fibroblast culture or lymphoblast for NP type C [6,19]. In this patient, an alteration in lipid metabolism was evident, and in the pathologic report of the spleen, there were macrophages with a wide and foamy cytoplasm, being negative for neoplastic and infectious findings, which was consistent with literature descriptions [16]. The beta-glycosidase reports showed an enzyme activity reduction. Nevertheless, it may have overexpression with high values like in the case herein. These results might be explained by the hereditary variation in each different case.

Among the therapeutic options, liver transplantation in the infant population for type A, and cordon cells or bone marrow transplantation for type B have been demonstrated. For type C, miglustat is demonstrated to improve the neurologic symptoms via the inhibition of glycosphingolipids biosynthesis, thereby reducing lipid storage, which has been demonstrated in clinical trials, showing improvement in symptoms in patients with mild to moderate neurological, psychiatric, or cognitive manifestations [20]. Although there is no specific treatment in our case, given the type of disease and pharmacologic indication, it should be important for health professionals to recognize this metabolic disorder and use a multidisciplinary approach, focusing on growth and developing assessments of nutritional state, lung functional status, and abdominal and neurologic surveillance. Furthermore, genetic counseling should be another factor taken into consideration, as despite its recessive autosomal features, each live birth has a 25% probability of being affected and 50% can be a carrier [20]. Pre-natal assessment tools are available for sphingomyelinase activity, which may be measurable from fibroblast and amniotic liquid or by a gene and molecular test [6].

## Conclusions

In conclusion, these types of diseases have a low frequency in the population, but it is not known whether it is a non-disease occurrence or whether it is a missed diagnosis. Considering how rare these storage diseases are, a differential diagnosis should be considered when other diagnosis options with similar symptoms and signs as splenomegaly and thrombocytopenia have been ruled out. Lastly, a neuropsychological follow-up should be made due to the frequency of complications among these patients.
